# Monitoring Temporal Trends in Cancer Survival; Choosing Appropriate Standards When Accounting for Age and Other-Cause Mortality Variation Over Time

**DOI:** 10.1158/1055-9965.EPI-24-1727

**Published:** 2025-07-01

**Authors:** Paul C Lambert, Therese M.-L. Andersson, Tor Åge Myklebust, Bjørn Møller, Mark J. Rutherford

**Affiliations:** 1 Department of Registration, Cancer Registry of Norway, Norwegian Institute of Public Health, Oslo, Norway; 2 Medical Epidemiology and Biostatistics, Karolinska Institutet, Stockholm, Sweden; 3 Department of Research and Innovation, Møre and Romsdal Hospital Trust, Ålesund, Norway; 4 Department of Population Health Sciences, University of Leicester, Leicester, UK

## Abstract

**Background:**

Along with incidence and mortality, temporal trends of cancer survival are a crucial part of cancer surveillance and control. The most common reported statistic is net survival, usually age standardized to an external reference population. However, net survival has an awkward interpretation, which has led to confusion and misunderstanding.

**Methods:**

We describe the use of reference adjusted all-cause survival and the crude probability of death as an alternative to net survival for the analysis of temporal trends in cancer survival. Reference adjusted measures aim to enable fair comparisons by incorporating additional reference expected mortality rates into the estimation process. The different approaches are illustrated using data on 95,285 women diagnosed with breast cancer in Norway 1986-2021.

**Results:**

We compare different age distributions for age standardization and describe how using a recent calendar period for both the reference expected mortality rates and age distribution for standardization leads to simple interpretation.

**Conclusions:**

Reference adjusted measures for monitoring temporal trends in cancer survival can lead to improved understanding and is of more relevance to patients and policy makers who live and make decisions in the real-world. Using the most recent calendar period for both the age standard and the reference expected mortality rates leads to simple and useful interpretation of the measures.

## Introduction

Population-based cancer registries play a critical role in cancer surveillance and control(1), enabling the monitoring and evaluation of temporal changes in incidence, mortality, and survival of different types of cancer. Cancer survival statistics help assess the effectiveness of health systems in the diagnosis and management of cancer(2,3). How to quantify temporal trends in cancer survival is the focus of the work here, but generally temporal trends in survival should be reported alongside trends in incidence and mortality(4,5).

In population-based cancer studies the most common reported statistic is net survival(6,7). The reason net survival is used extensively is that it enables comparisons of survival over time, or between population groups, that are independent of rates of death due to other causes, which usually vary over calendar time and between population groups. The interpretation of net survival is somewhat awkward in that is gives the probability of being alive as a function of time after a diagnosis of cancer, *if it was impossible to die from anything other than the cancer under study*. Net survival is thus a hypothetical measure as individuals diagnosed with cancer will always be at risk of death from other causes.

Two alternative metrics for quantifying survival are the all-cause probability of death/survival and the crude probability of death(3,8,9), which give the probability of dying/surviving from any cause and the probability of dying from the cancer of interest, respectively. These “real-world” measures are useful for understanding the impact on the population and have a simpler interpretation than net survival. However, they are not usually advocated for temporal comparisons as they do not isolate cancer mortality rates from other cause mortality rates. For example, if there are changes in all-cause survival or the crude probability of death over calendar time, these changes could be due to differences in cancer mortality rates, differences in other cause mortality rates or some combination of both.

Reference adjustment is a recent proposal to enable comparisons of all-cause survival and/or crude probabilities of death over time or between population groups in such a way that any differences will be due to differential cancer mortality rates and not due to differences in other cause mortality rates(10,11). This is achieved by defining reference (common) expected mortality rates that are fixed over time, in addition to the actual expected mortality rates that vary over calendar time. Both the actual and reference expected mortality rates used in the estimation of the measures with estimates giving what the probability would be in a population with the reference population other cause mortality rates. There are different choices for the reference expected rates, which we will discuss.

An additional issue when investigating temporal trends is age standardization(12,13). The age distribution usually changes over calendar time and age standardization is used to force the same age distribution on all calendar periods being compared. This means there is a choice over which age distribution is used and in practice different age standards are used in different studies. A common choice is International Cancer Survival Standard (ICSS) age distribution(14), but this age distribution may be very different from that observed in a particular geographical area and it may be more relevant to choose an age distribution that is closer to the population under study.

In this paper we describe and advocate the use of reference adjusted measures in monitoring temporal trends in cancer survival as a complement/alternative to net survival. We define the various measures and how reference adjustment can be incorporated when estimating all-cause survival and the crude probability of death. We also discuss how careful choice of both the reference expected rates and the age distribution for standardization can lead to estimates of the real-world experience of the population of most interest. We provide Stata code to show how the methods can be implemented.

## Materials and Methods

We first describe the different measures of survival with a focus on their use in assessing temporal trends.

### All-Cause Probability Of Death/Survival

All-cause survival is the probability of being alive as a function of time since diagnosis. It does not distinguish between deaths due to the cancer of interest or from other causes. Although it is possible to investigate trends of all-cause survival over calendar time, for example using Kaplan-Meier estimates, there are two main. Firstly, there are changes in other cause mortality rates over calendar time and even if there were no changes in cancer-specific mortality rates, one would usually expect to see improvements in all-cause survival. Secondly, the age distribution is likely to change over time and thus any differences could be partly due to a shifting age distribution, particularly for long-term trends. Age standardization could be used to remove differences due to the age distribution, but differences due to changes in other cause mortality rates would remain.

### Crude Probability of Death Due To Cancer

The crude probability of death due to cancer is the probability of dying from the cancer accounting for the fact that some individuals may die of other causes(9,15). When using crude probabilities, the probability of death due to all causes is partitioned into that due to the cancer and that due to other causes. Crude probabilities are a function of both cancer mortality rates and other cause mortality rates. For example, consider a 50-year-old and an 80-year-old with exactly the same cancer mortality rates; the 80-year old would have a lower crude probability of death due to cancer as they would be more likely to die from other causes. It has been advocated that crude probabilities are more relevant to patients than net survival(3,15). However, they suffer from the same disadvantages as all-cause survival in that any differences could be due to differences in cancer mortality rates, differences in other cause mortality rates or a combination of both.

### Net Survival

Net survival is the probability of being alive as a function of time since diagnosis in the hypothetical situation where it is impossible to die from any cause other than the cancer under study. Net survival is the most common way of investigating trends in cancer survival over calendar time as it attempts to remove differences in other-cause (other cause) mortality rates. Although net survival has a somewhat awkward interpretation due to the hypothetical situation of not dying from other causes, it provides a measure where any differences should only be due to differences in cancer mortality rates. It has been suggested that survival measures should “stick to this world”(16), but, as discussed above, “real-world” measures do not isolate cancer specific mortality rates. For comparisons over calendar time age standardization is performed due to a changing age distribution. It is common to age standardize using the ICSS, but as explained below, this age standard may be very different to the observed age distribution.

### Relative Survival Versus Cause-Specific Survival

In population-based cancer studies rather than relying on cause of death information both net survival and the crude probability of death are usually estimated in the relative survival framework(6). Cause-specific survival uses information obtained from death certificates to determine whether a death was due to the cancer or not. However, using cause of death information is potentially problematic due to misclassification of cause of death(17) or not capturing deaths indirectly due to the cancer(18). In the relative survival framework, the mortality rate in excess of that expected in a similar group in the general population is estimated with expected mortality rates obtained from lifetables stratified by age, calendar year and sex. Other relevant factors could be included if available (e.g. region or socio-economic group). An important assumption in the relative survival framework is that the expected mortality rates reflect the other cause mortality rates of the study population(19).

We now describe reference adjusted measures, which are calculated in the relative survival framework.

### Reference Adjusted All-Cause Survival

The idea behind reference adjusted all-cause survival is to adapt all-cause survival so that any differences between population groups are solely due to differences in cancer mortality rates, and therefore not due to any potential differences in other cause mortality rates. Reference adjusted all-cause survival gives the all-cause survival that would be observed for the cancer cohort if they instead experienced a common reference standard expected mortality rate, rather than the rate currently assumed for the cohort. First, other cause mortality is removed by calculating relative survival using the relevant expected rates for the cohort under study. We then convert back to an all-cause measure using the reference expected mortality rates, which are common across the compared groups. Combining reference adjustment with a sensible choice for the age distribution to standardize to, leads to simpler interpretation.

Below we give the conceptional idea of the approach and refer to Lambert *et al*.(10) and Rutherford *et al*.(11) for further technical details of the model based and non-parametric approaches respectively.

The relative survival framework is used, so the all-cause survival, *S*_*ij*_(*t*), for an individual, *i*, diagnosed in calendar period, *j*, is expressed as the product of the expected survival, $S_{ij}^{*}(t)$, and the relative survival *R*_*ij*_(*t*).

1 $$S_{ij}(t)=S_{ij}^{*}(t)R_{ij}(t)$$

Under assumptions, *R*_*ij*_(*t*), can be interpreted as net survival(7). The terms in the above equation have *i* and *j* subscripts as both expected and relative survival vary between individuals and over calendar time.

The aim of reference adjustment for temporal trends is to incorporate expected survival, $S_{i}^{**}(t)$, that is common over calendar time, so that the reference adjusted all-cause survival, $S_{ij}^{R}(t)$, is expressed as.

2 $$S_{ij}^{R}(t)=S_{i}^{**}(t)R_{ij}(t)=\frac{S_{i}^{**}(t)S_{ij}(t)}{S_{ij}^{*}(t)}$$

For comparisons over calendar time, the key point is that expected survival for a specific age is common for all calendar periods. This means that any differences will only be due to differential relative survival, *R*_*ij*_(*t*). If $S_{ij}^{**}(t)=S_{i}^{*}(t)$ for one calendar period, then the reference adjusted all-cause survival is identical to all-cause survival. It is important to note that the appropriate expected survival for a particular population, $S_{ij}^{*}(t)$, is used in the estimation of *R*_*ij*_ (*t*).

We generally want to obtain the average (marginal) survival for each calendar period as a summary measure. This is the average $S_{ij}^{R}(t)$ over the *N*^*j*^ individuals in calendar period *j*.

3 $${\bar{S}}_{j}^{R}(t)=\frac{1}{N_{j}}\sum_{i=1}^{N_{j}}S_{ij}^{R}(t)$$

For comparisons we also need to standardize to a common age distribution. This can be done by estimating survival separately in age groups and then obtaining a weighted average (traditional age standardization) or by introducing individual weights, *w*_*ij*_,

4 $${\bar{S}}_{j}^{R}(t)=\frac{1}{N_{j}}\sum_{i=1}^{N_{j}}w_{ij}S_{ij}^{R}(t)$$

This up-or down- weights individuals relative to a reference population(20). We have adopted the individual weighting approach for age standardization as it is useful in smaller study populations(13). The choice of reference age distribution and reference expected rates is discussed below.

### Reference Adjusted Crude Probabilities of Death Due To Cancer

The same ideas for reference adjusted all-cause survival described above, i.e. introducing reference weights, can be applied to the crude probability of death due to cancer. Reference adjusted crude probabilities of death due to cancer or other causes gives probabilities that would be observed for the cancer cohort if they instead experienced the reference expected mortality rates.

The crude probability of death due to cancer for the *i*^*th*^ individual in the *j*^*th*^ calendar period is defined as,

$$CP_{C,ij}(t)=\int_{0}^{t}S_{ij}^{*}(u)R_{ij}(u)\lambda_{ij}(u)du$$

where *λ*_*ij*_ (*t*) is the excess mortality rate.

Similarly, the crude probability of death due to other causes is defined as,

$$CP_{O,ij}(t)=\int_{0}^{t}S_{ij}^{*}(u)R_{ij}(u)h_{ij}^{*}(u)du$$

Where $h_{ij}^{*}(u)$ is the expected mortality rate.

For reference adjustment we replace $S_{ij}^{*}(u)$ with $S_{ij}^{**}(u)\;$ and $h_{ij}^{*}\;$ with $h_{ij}^{**}$ The reference adjusted crude probability of death due to cancer is,

$$CP_{C,ij}^{R}(t)=\int_{0}^{t}S_{ij}^{**}(u)R_{ij}(u)\lambda_{ij}(u)du$$

The reference adjusted crude probability of death due other causes.

$$CP_{O,ij}^{R}(t)=\int_{0}^{t}S_{ij}^{**}(u)R_{ij}(u)h_{ij}^{**}(u)du$$

As for reference adjusted all cause survival, the crude probabilities can be age standardized through incorporation of individual weights. The model based estimates of reference adjusted crude probabilities use the equations above, see Lambert *et al*(10). The non-parametric estimator does not directly estimate the individual contributions, but, through incorporation of inverse probability weights, obtains the marginal estimates directly, see Rutherford *et al*. for details(11).

### Choice of Age Distribution For Age Standardization

Two potential choices for the age distribution to standardize over are,

1. An age distribution from a (hypothetical) external population, such as (ICSS)(14).
2. An age distribution for a particular group in the study population.

The advantage of using a standard such as ICSS is that the study is comparable to other studies using the same standard. The disadvantage is that the reference age distribution may be very different to that observed in the study population. For example, in Europe many cancers have an older age at diagnosis than the ICSS age distribution, resulting in estimated survival for a hypothetical population that is much younger than that observed.

When looking at temporal trends within a particular geographical area, choosing the most recent calendar year/period as the reference means that the most current estimates reflect the current population. This is the approach used in the annual report of the cancer registries in Norway(21) and Finland(22), whilst others such as England(23) and SEER in the USA(24) use the ICSS. A key advantage of using the last calendar period is that estimates are more reflective of those diagnosed recently. One disadvantage of choosing a recent calendar period is that in subsequent years the estimates for a particular calendar period may change as the age standard would change to a new age distribution.

### Choice of Reference Population For Expected Survival

Similar to age standardization, the reference population could be chosen to be a common standard, which would ensure comparability between different studies that use this standard (and the same age distribution for standardization). For example a set of papers comparing survival in the Nordic countries used the average expected survival across the Nordic countries to define the reference population(25–27).

When investigating temporal trends, choosing the most recent calendar year/period for both the reference expected survival, and the age distribution to standardize to, ensures that the most current estimates reflect the current factual population survival estimates, and prior calendar periods are therefore adjusted to ensure fair comparisons over calendar time.

### Estimation

Reference adjusted estimates can be obtained using either a modelling(10) or non-parametric framework(11). In the modelling framework a statistical model is fitted and a predicted reference adjusted survival curve for each individual obtained using equation (2) with the marginal (average) estimate obtained using equation (3) or the age standardized estimate obtained using equation (4). In the non-parametric setting, the marginal (average) estimate is directly estimated, through incorporation of weights based on the ratio of the reference and observed expected survival functions combined with additional weights for age standardization. We adopt the non-parametric approach here and provide Stata code in the Supplementary Materials and Methods. See the papers of Lundberg *et al*.(25–27) for examples of the statistical modelling approach.

### Example

We illustrate the various measures using Breast cancer survival for women diagnosed in Norway between 1986 and 2021. All diagnoses of women with breast cancer in the Cancer Registry of Norway were identified. Cancer cases diagnosed incidentally at autopsy were excluded, and only the first recorded breast cancer diagnosis per individual was considered. The final study population consisted of 95,285 women. Individuals were followed from the date of diagnosis until death or emigration or the end of the follow-up period which was December 31, 2021. Mortality rates in the Norwegian general population were stratified by age, year, and sex.

For the various measures we compared 7 calendar periods (1986-1990, 1991-1995, 1996-2000, 2001-2005, 2006-2010, 2011-2015, and 2015-2021). We compare 5-year marginal net survival, all-cause survival and the crude probability of death over calendar time. We compare unadjusted estimates to age standardization using the ICSS age distribution and the age distribution in the last calendar period. For all-cause survival and the crude probability of death we additionally use reference adjustment with the reference expected survival calculated as the average over the six years in the last calendar period.

All analysis were performed at the Cancer Registry of Norway (CRN), which is statutory, and statistics were made available according to the Norwegian Health Register Act §19. Ethical approval was not required for the produced statistics.

## Results

**Figure 1:**
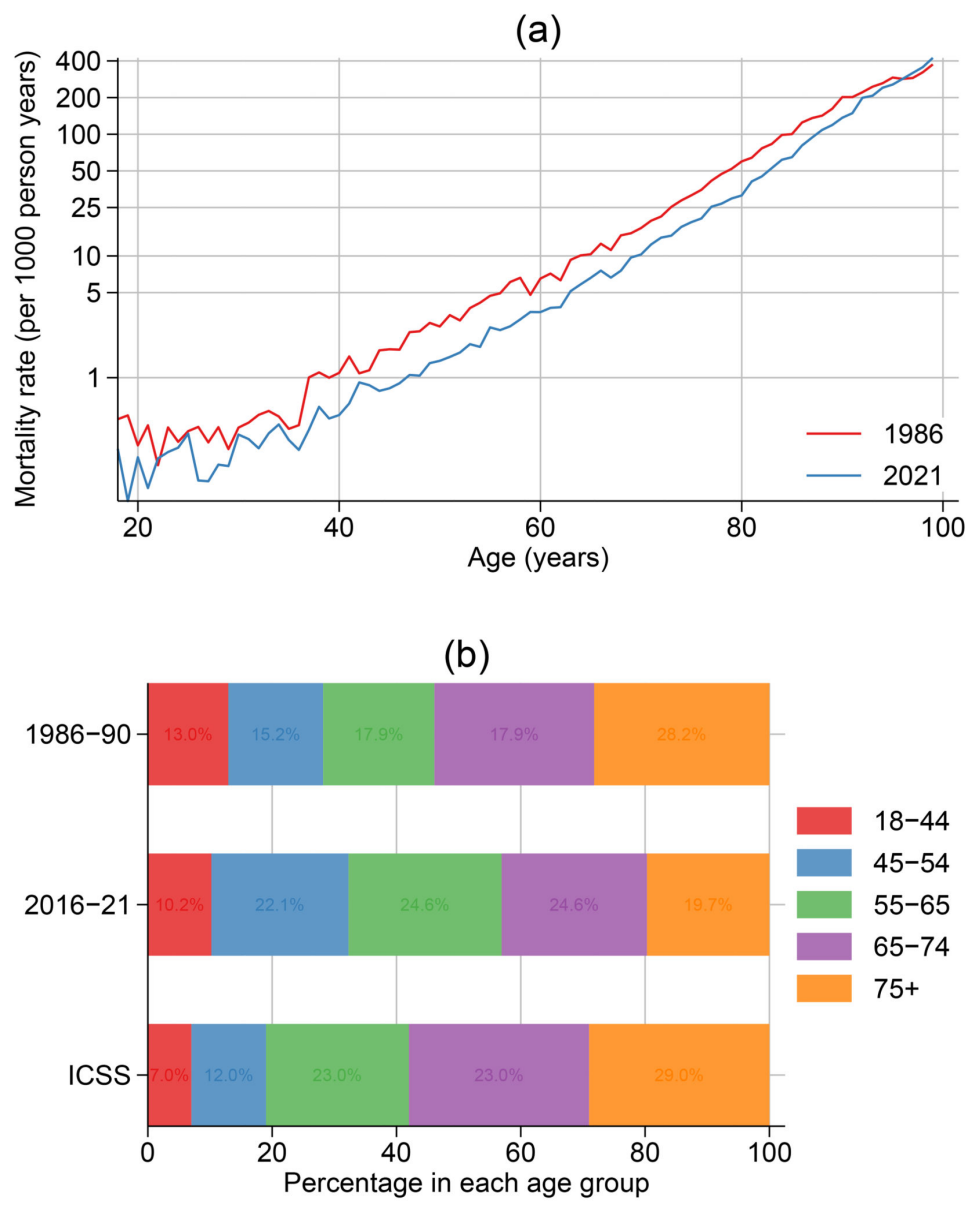
Changes in expected mortality rates and the age distribution at diagnosis for women diagnosed with breast cancer in Norway 1986-2021. (a) Mortality rates in Norway in 1986 and 2021 for females aged 18-99. (b) Age distribution for the International Cancer Survival Standard (ICSS) age groups and for women diagnosed with breast cancer in Norway in 1986-1990 and 2016-2021

Figure 1a shows the mortality rates for females ages 18-99 in the Norwegian general population at the beginning (1986) and end (2021) of the study period. There has been a clear reduction in mortality rates over all ages except for the very oldest individuals. Figure 1b shows the age distribution for the first (1986-1990) and last (2016-2021) calendar periods together with the ICSS age distribution. Within Norway there has been a noticeable shift in the age distribution at diagnosis, with a decrease from 28.2% diagnosed at age 75+ in 1986-1990 to 19.7% in 2016-2021. The ICSS age distribution was broadly similar to the age distribution in Norway in 1986-1990, but in the most recent calendar period there are notably higher proportions diagnosed in age groups, 18-44, 45-54, and 55-64. This means that when using the ICSS age distribution for the most recent calendar period an older age distribution is forced on the study population and thus we expect survival to be lower. Choosing the most recent calendar period for both the expected mortality rates and the age distribution for standardization will lead to factual estimates for the most recent calendar period.

**Figure 2:**
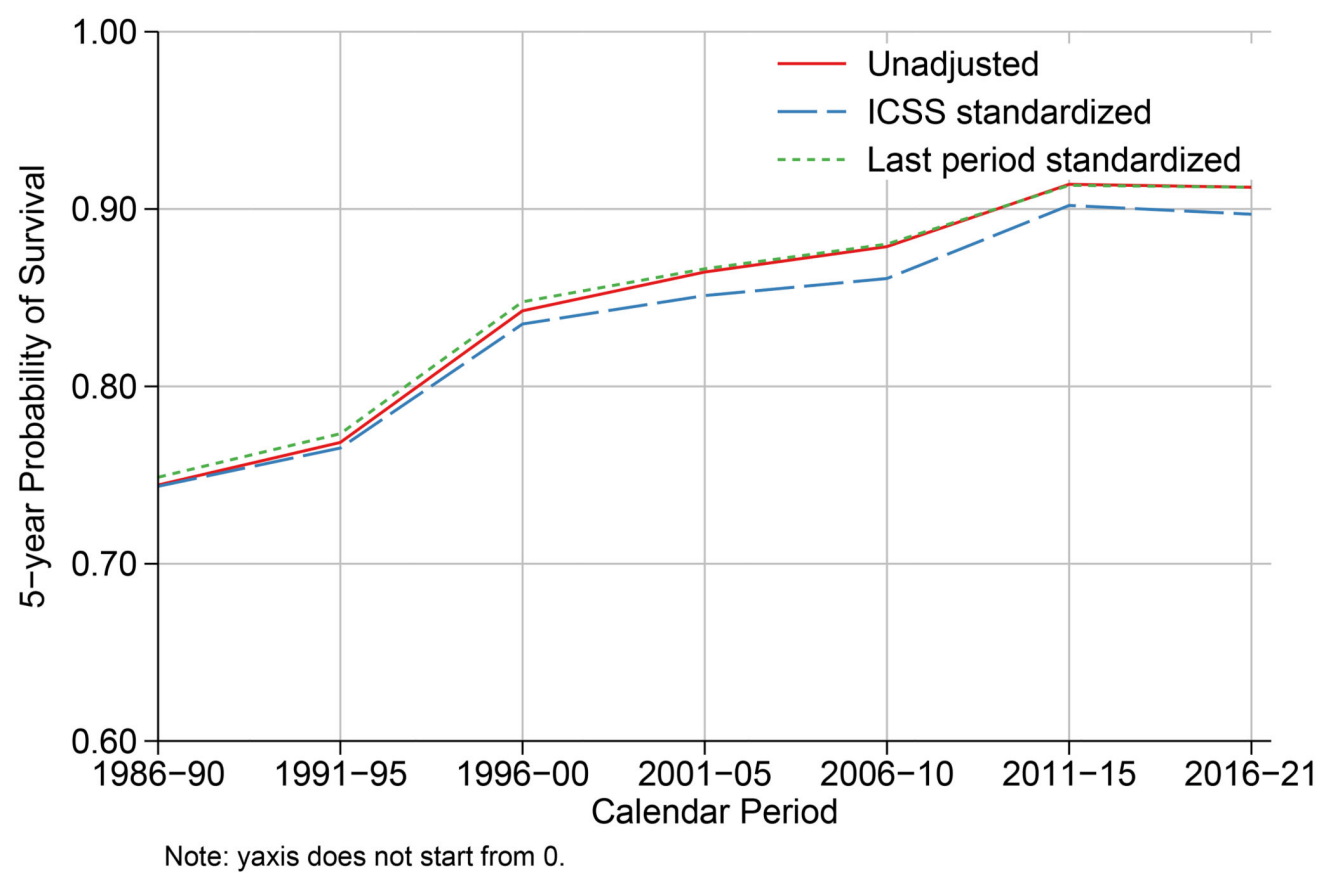
Trends in net survival for women diagnosed with breast cancer in Norway 1986-2021. Estimates are unstandardized, age standardized to the last calendar period (2016-2021) or to the ICSS age distribution.

Figure 2 shows temporal trends in net survival between 1986 and 2021 comparing unstandardized estimates with standardized estimates using both the ICSS age distribution and the age distribution in last calendar period (2016-2021). When age standardizing to the age distribution in last calendar period, the estimates are identical to the unadjusted estimates for this last calendar period. The age standardized estimates for earlier calendar periods are slightly higher than the unadjusted estimates as they are being standardized to a population younger than observed. When standardizing using the ICSS age standard, survival is lower than that observed. This is because an older population is used as the age standard and net survival is lower among the older age groups.

**Figure 3:**
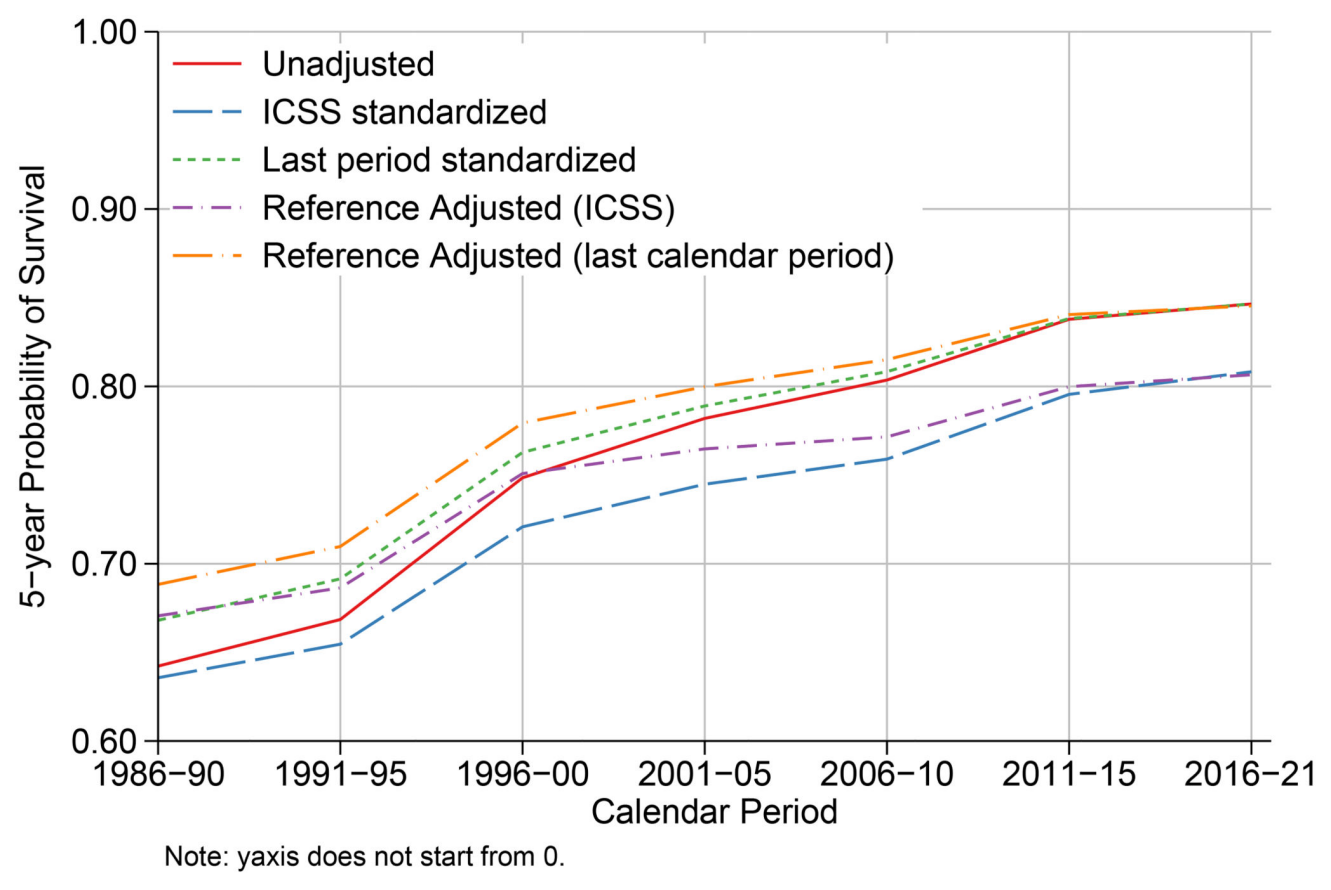
Trends in all-cause survival for women diagnosed with breast cancer in Norway 1986-2021. Estimates are unstandardized, age standardized to the last calendar period (with and without reference adjustment) or age standardized to the ICSS age distribution (with and without reference adjustment).

Figure 3 shows temporal trends in all-cause survival 1986-2021 comparing unstandardized estimates with standardized estimates, using both the last calendar period (2016-2021) and the ICSS age distribution, together with reference adjusted estimates. Survival is lower than net survival (Figure 2) as all-cause survival incorporates deaths due to other causes. When age standardizing to the age distribution in the last calendar period, the estimates are the same as the unstandardized estimates for this last calendar period. The standardized estimates for earlier calendar periods are higher than unadjusted estimates as they are standardized to a population younger than observed. However, this is not a fair comparison over calendar period as expected survival changes over calendar time. This is solved by using reference adjustment where the reference rates are the expected rates are for the last calendar period. The estimate for the last calendar period remains a factual estimate, with previous calendar periods adjusted for changes in both other cause mortality rates and the age distribution. There are very small differences when using reference adjustment compared to the unadjusted values in the last calendar period as the reference rates are averaged over the calendar period, whilst unadjusted estimates use separates rates for each year. There are notable differences when using ICSS weights for age standardization as the estimates are for an older population than actually observed in Norway.

**Figure 4:**
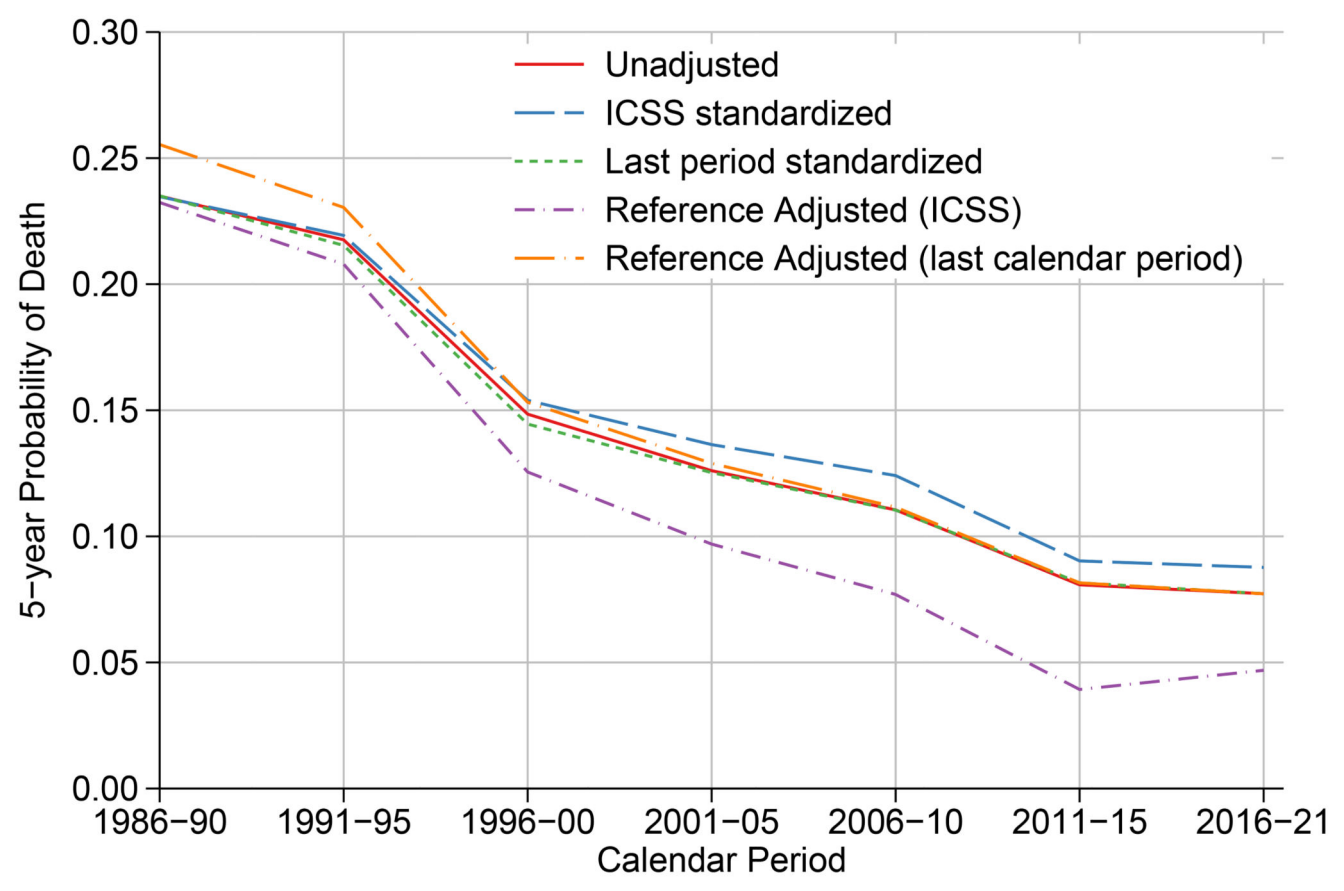
Trends in the crude probability of death for women diagnosed with breast cancer in Norway 1986-2021. Estimates are unstandardized, age standardized to the last calendar period (with and without reference adjustment) or age standardized to the ICSS age distribution (with and without reference adjustment).

**Figure 5:**
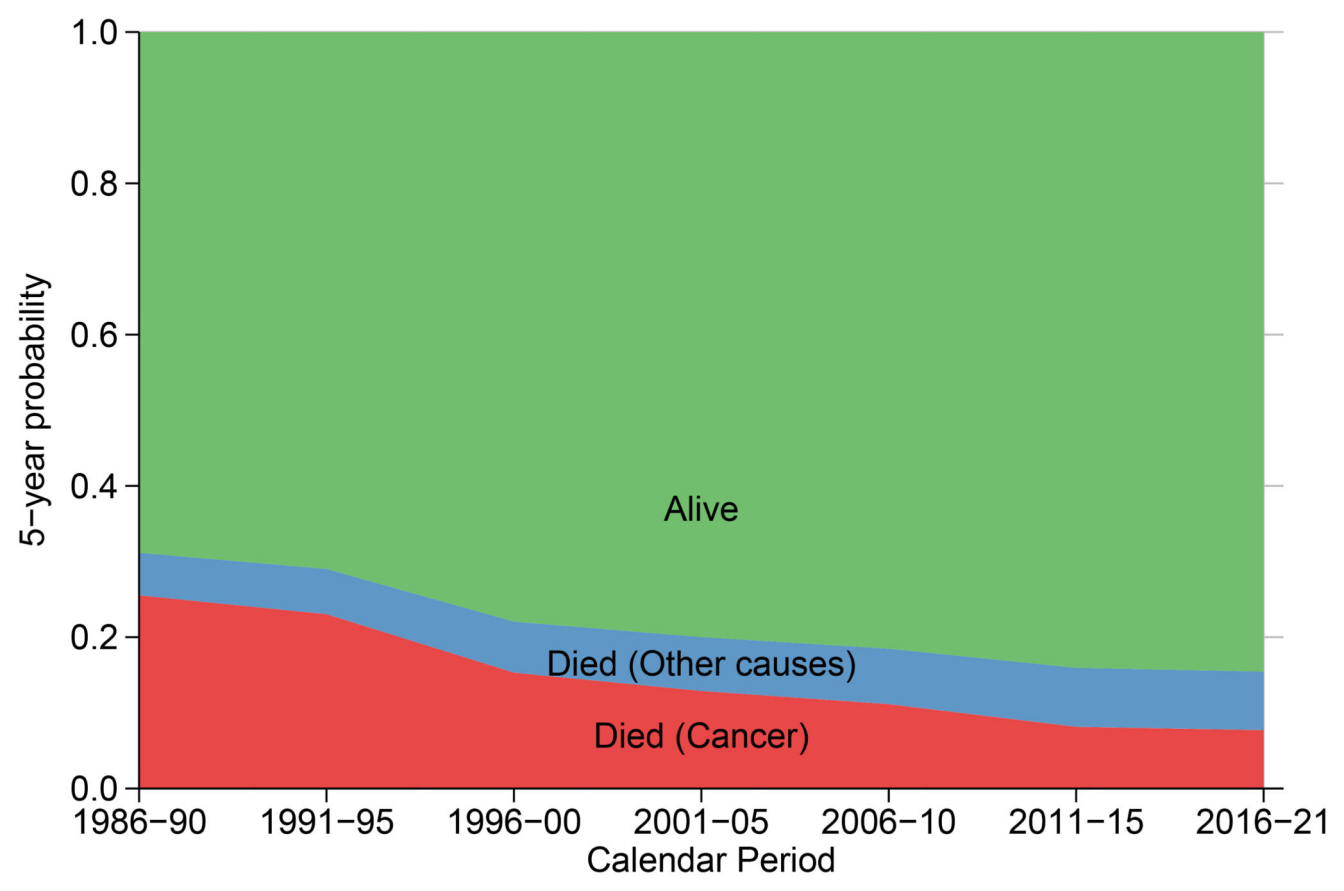
Stacked graphs showing the probability of being alive, dying from cancer and dying from other causes for women diagnosed with breast cancer in Norway in 1986-2021. Estimates are age standardized to the age distribution in 2016-2021 with reference adjustment to the last calendar period.

Figure 4 shows temporal trends in the crude probability of death 1986-2021 comparing unadjusted with standardized estimates using both the last calendar period (2016-2021) and the ICSS age distribution together with reference adjusted estimates. There are similar patterns to all-cause survival and notably ICSS weights give a higher probability of death due to the older age distribution. The reference adjusted estimates using the age distribution for the last calendar period give factual estimates for this calendar period. Figure 5 shows crude probabilities in a stacked graph which partitions the probability of dying from any cause into that due to cancer and that due to other causes, whilst also showing the probability of being alive. Estimates have been age standardized to the last calendar period and reference adjusted to the expected survival in the last calendar period. This means that the differences observed in the figure are only due to differential other cause mortality, whilst the estimates for the last calendar period reflect what was observed in that period.

## Discussion

This paper has illustrated the use of reference adjustment when quantifying temporal changes in cancer survival. When comparing cancer survival over calendar time, it is important that differences are solely due to differences in cancer-specific mortality rates (here estimated in the relative survival framework), and not contaminated by differences in a changing age distribution or due to different expected mortality rates. This is why, despite its interpretational awkwardness, ICSS age standardized relative survival has remained the metric of choice in many population-based cancer studies. However, it is of less relevance to patients or policy makers who live or have to make decisions in the real-world(28). Reference adjusted measures provide an alternative and, with appropriate choice of the age standard for standardization and reference expected mortality rates, may lead to better understanding of temporal changes in survival.

There are many examples of net survival being misunderstood and misinterpreted with it sometimes being interpreted as all-cause survival. Interpretation is simpler for both all-cause survival and the crude probability of death and both measures retain information about the overall burden of mortality through incorporation of deaths both due to cancer and other causes. However, to make fair comparisons, for all but one group, estimates must be hypothetical in that both the reference expected rates and the age distribution for standardization will only apply exactly to one group. For temporal trends the most recent calendar period is a sensible choice for both the age distribution and the reference mortality rates. Net survival is a special case of reference adjusted survival where the reference expected rates are forced to be the same, i.e. the reference expected survival probability is 1 for all individuals at all times.

It is essential to age standardize when comparing survival over calendar time or between population groups. Age standardization generally leads to hypothetical measures as a different age distribution to that observed is forced on a population. Some cancer registries, including Norway and Finland, use a recent calendar period as the age standard with a separate age standard for each cancer site to reflect different age distributions. This means that estimates for the last calendar period reflect what was observed in that period and therefore no longer are hypothetical. When combined with using reference expected mortality rates from the last period the all-cause survival or crude probability of death is identical to that observed in that calendar period. This approach was implemented in a recent report in Norway(29). Other registries choose to use ICSS weights. Although we see the advantage of comparability between countries through using ICSS weights, this is often not the main purpose of national reports. The ICSS age distribution can be very different from observed; for example, in Norway in 2016-2021 for Bladder cancer the observed percentages in the 5 ICSS age groups was 0.5%, 3.6%, 13.8%, 30.9% and 51.2% compared to the ICCS percentages of 7.0%, 12.0%, 23.0%, 23.0% and 29.9% respectively.

One disadvantage of not using the ICSS age distribution for standardization is that results will not be directly comparable between cancer sites or between countries using a different age standard. This raises the question of who the analysis is for? For studies comparing survival between countries, (e.g. CONCORD(30) or International Cancer Benchmarking Partnership studies(31)), it is essential that a common age standard is used, with the ICSS age distribution being applied. However, for understanding survival in a particular country, it is more appropriate to use a relevant, up-to-date age distribution. Thus, the choice of age distribution for standardization should depend on the purpose of the analysis. For example, for comparisons between countries if the viewpoint is for a particular country and how its survival compares to others, it would be appropriate to use that country’s recent observed age distribution for standardization and, if wanting to report all cause survival or the crude probability of death, using recent expected mortality rates for that country.

We have used non-parametric estimation of reference adjusted measures in this paper as we feel this approach would be easier for cancer registries to implement. However, it is possible perform estimation in a modelling framework(10), with the advantage that other measures such as reference adjusted loss in life expectancy(32) can be calculated.

In summary, we have presented an alternative method to net survival to present temporal trends in cancer survival, which can lead to improved understanding by providing metrics closer to the real world.

## Supplementary Material

1

## Data Availability

This study used data from the Cancer Registry of Norway, which is available upon application at helsedata.no.

## Impact

Increasing the use of reference adjusted measures in the analysis of population-based cancer studies will enhance understanding of cancer survival trends. The freely available software increases the likelihood of uptake.
