# Supplementary material for: Monitoring Temporal Trends in Cancer Survival; Choosing Appropriate Standards When Accounting for Age and Other-Cause Mortality Variation Over Time
Source: Cancer Epidemiol Biomarkers Prev. Author manuscript; Available in PMC 2025 Sep 9. (PMC7618090; doi:10.1158/1055-9965.EPI-24-1727)
Supplement: 1 [file EMS204886-supplement-1.pdf]

Supplementary Materials and Methods: Stata code

```
clear frames
frames reset

global root <root folder>

global POPMORT <location of population mortality file>

// create reference adjusted popmort file
// average over last calendar period
use $POPMORT, clear
keep if inlist(_year,2016,2017,2018,2019,2020,2021)
collapse (mean) prob, by(sex _age)
gen rate_lt=-ln(prob)
save ${root}/Data/popmort_refadj, replace
global POPMORT_refadj ${root}/Data/popmort_refadj.dta

// Load data
use ${root}/Data/breast_2021, clear
stset exitdate, origin(diagdate) failure(dead=1) scale(365.24) ///
                exit(time diagdate + 6*365.25) id(id)

// generate individual weights using ICSS1 reference
genindweights indwt_ICSS, by(calperiod sex) agegroup(ICSSagegrp)
refexternal(ICSS1_5)

// generate individual weights where reference is the
// last calendar period (use 10 age groups).
// create 10 agegroups
egen agegroup10 = cut(agediag), group(10) icodes
genindweights indwt_lp, by(calperiod sex) ///
                        refconditional(calperiod==7, strata(agegroup10))
///
                        refproportion(ref_wt_last_period)

// check reference weights calculated correctly
bysort calperiod sex agegroup10: gen first=_n==1
list sex agegroup10 ref_wt_last_period if calperiod==7 & first, noobs
tab agegroup10 if calperiod==7

// Unadjusted
stpp RS1 using $POPMORT,                 ///
              agediag(agediag)           ///
              datediag(diagdate)         ///
              pmother(sex)               ///
              pmrate(rate_lt)            ///
              list(1 5)                  ///
              by(calperiod sex)          ///
              allcause(AC)               ///
              crudeprob(CPc CPo)         ///
              frame(unadjusted, replace)
```

```
// ICSS age standardized
stpp RS2 using $POPMORT,              ///
              agediag(agediag)        ///
              datediag(diagdate)      ///
              pmother(sex)            ///
              pmrate(rate_lt)         ///
              list(1 5)               ///
              by(calperiod sex)       ///
              allcause(AC2)           ///
              crudeprob(CP2c CP2o)    ///
              indweights(indwt_ICSS)  ///
              frame(ICSS, replace)

// Last Period age standardized
stpp RS3 using $POPMORT,              ///
              agediag(agediag)        ///
              datediag(diagdate)      ///
              pmother(sex)            ///
              pmrate(rate_lt)         ///
              list(1 5)               ///
              by(calperiod sex)       ///
              allcause(AC3)           ///
              crudeprob(CP3c CP3o)    ///
              indweights(indwt_lp)    ///
              frame(lastper, replace)

// Reference adjusted (ICSS age weights)
stpp RS4 using $POPMORT,              ///
              agediag(agediag)        ///
              datediag(diagdate)      ///
              pmother(sex)            ///
              pmrate(rate_lt)         ///
              list(1 5)               ///
              by(calperiod sex)       ///
              allcause(AC4)           ///
              crudeprob(CP4c CP4o)    ///
              indweights(indwt_ICSS)  ///
              using2($POPMORT_refadj, ///
                     pmyear2(.))      ///
              frame(ICSS_refadj, replace)

// Reference adjusted (last period age weights)
stpp RS5 using $POPMORT,              ///
              agediag(agediag)        ///
              datediag(diagdate)      ///
              pmother(sex)            ///
              pmrate(rate_lt)         ///
              list(1 5)               ///
              by(calperiod sex)       ///
              allcause(AC5)           ///
              crudeprob(CP5c CP5o)    ///
              indweights(indwt_lp)    ///
              using2($POPMORT_refadj, ///
                     pmyear2(.))      ///
              frame(lastper_refadj, replace)
```
